# Peer-Reviewed Reflective Writing by Phase II Medical Students: A Mixed-Method Study

**DOI:** 10.7759/cureus.90679

**Published:** 2025-08-21

**Authors:** Abiselvi Annasekaran, V Rajasekar, Rajavel Murugan P, V Kalaivani

**Affiliations:** 1 Department of Community Medicine, Government Thoothukudi Medical College, Thoothukudi, IND; 2 Departmentt of General Medicine, Government Thoothukudi Medical College, Thoothukudi, IND; 3 Department of Microbiology, Government Thoothukudi Medical College, Thoothukudi, IND

**Keywords:** collaborative clinical thinking, gibbs reflective writing, medical students, peer review, reflective writing

## Abstract

Background: Reflective writing is a critical educational tool in medical training, promoting self-awareness, critical thinking, and professional growth. Peer review adds a collaborative dimension to reflective practices, enhancing depth and clarity through constructive feedback. However, challenges such as limited guidance, time constraints, and language barriers may hinder its effectiveness. This study evaluated the impact of peer-reviewed reflective writing on second-year medical students at Government Thoothukudi Medical College, India.

Methods: A mixed-methods study was conducted with 30 second-year medical students enrolled in a Community Medicine block posting. Training sessions introduced students to Gibbs' Reflective Cycle and peer review principles using a standardized rubric. Students wrote initial reflections, participated in peer review, and revised their submissions based on feedback. Quantitative data from pre- and post-intervention surveys and rubric-based assessments of reflective writings were analyzed for improvements. Focus group discussions (FGDs) provided qualitative insights into students' experiences.

Results: Thirty students participated in the study. Quantitative analysis showed significant improvements in perceived value (3.2 ± 0.9 to 4.5 ± 0.7, p < 0.001) and confidence in reflective writing (2.8 ± 1.0 to 4.3 ± 0.8, p < 0.001). Reflective writing quality improved in clarity (3.1 ± 0.8 to 4.6 ± 0.5), depth (2.9 ± 1.0 to 4.4 ± 0.6), and adherence to Gibbs' Cycle (3.0 ± 0.7 to 4.7 ± 0.4; p < 0.001). FGDs revealed increased reflective depth, enhanced peer collaboration, and challenges such as language barriers and time constraints.

Conclusion: Peer-reviewed reflective writing significantly enhances reflective practices, fostering critical thinking and professional growth. Addressing challenges, such as language barriers and variability in feedback quality, can further optimize outcomes. Integrating structured peer review into medical curricula can prepare students for reflective, collaborative clinical practice.

## Introduction

Reflective practice is a cornerstone of medical education, fostering critical thinking and self-awareness among medical students [1]. Among various educational strategies, peer review has emerged as a potent tool to enhance reflective writing skills. It promotes collaborative learning, constructive feedback, and mutual improvement, which are essential in cultivating the competencies required for patient-centered care [2]. Phase II medical students (second professional), who are transitioning from preclinical to clinical environments, are particularly poised to benefit from these activities as they develop their professional identities and clinical reasoning [3]. Reflective writing is not only a pedagogical exercise but also a medium for students to process their experiences, emotions, and insights during medical training. Peer review adds another dimension to this process by incorporating diverse perspectives, enabling students to identify blind spots, and encouraging deeper engagement with reflective narratives [4]. The value of peer review lies in its ability to simulate real-world clinical practices, where teamwork and feedback are integral to effective decision-making and professional growth [5].

This study explores the experiences of Phase II medical students engaging in peer review of reflective writing of the context taught during their clinical postings. By employing a qualitative approach, it seeks to understand how peer review influences their reflective writing processes and how it shapes their learning experiences. The findings aim to contribute to the growing body of evidence supporting reflective practices in the context learned in medical education, offering insights into the dynamics of peer interactions and their impact on reflective learning.

The integration of reflective practices in medical education is grounded in theories of experiential learning, which emphasize the iterative process of reflecting on experiences to gain insights and foster professional growth [6]. Reflective writing, in particular, has been widely adopted as an educational tool to facilitate this process, helping students articulate their thoughts, analyze their experiences, and connect theory to practice [7]. However, despite its benefits, many students struggle with reflective writing due to a lack of structured guidance, limited feedback, and uncertainties about expectations [8]. Peer review provides a mechanism to address these challenges by creating a supportive environment where students can critique and learn from each other’s reflective narratives. Studies have shown that peer review fosters critical appraisal skills, encourages active engagement, and enhances students' ability to construct and refine their reflective work [9,10]. Furthermore, the collaborative nature of peer review aligns with the interprofessional and teamwork-oriented competencies emphasized in medical education frameworks [11].

Phase II medical students, at a pivotal stage in their training, face increasing clinical responsibilities and complex learning environments. Reflective writing becomes particularly important during this phase, as it allows students to process the emotional and cognitive challenges of clinical practice, understand their professional roles, and navigate ethical dilemmas [12]. Peer review serves as a complementary strategy, encouraging these students to deepen their reflections, recognize different viewpoints, and develop skills in providing and receiving constructive feedback [13].

The rationale for this study stems from the limited qualitative research exploring the experiences of medical students with peer review of reflective writing. While existing studies have highlighted the benefits of reflective writing and peer review independently, there is a paucity of evidence on how these two practices intersect and influence student learning, particularly in the context of medical education [14]. By focusing on Phase II medical students, this research aims to address this gap, providing insights into how peer review can be optimized to support reflective practices and contribute to the holistic development of future healthcare professionals.

Objectives of this study were (a) to explore the perceptions of medical students towards peer-reviewed reflective writing using Gibbs' Reflective Cycle, (b) to assess the impact of peer review on the quality and depth of reflective writing among medical students, and (c) to identify any challenges or barriers students face during the peer review and suggest improvements.

## Materials and methods

Study design and participants

A convergent mixed-methods study was conducted, integrating both quantitative and qualitative approaches, to evaluate the impact of peer-reviewed reflective writing on medical students' learning experiences. The participants included 30 second-year medical students enrolled in the Community Medicine Clinical Posting course at Government Thoothukudi Medical College. The study was approved by the Institutional Ethics Committee (IEC), Reference Number 03/2024-02 at Government Thoothukudi Medical College. Participation was voluntary, and all participants provided informed consent before participating in the study. 

Procedure

Training session: A workshop was conducted at the outset of the study to introduce students to Gibbs' Reflective Cycle and the principles of effective peer review. During the session, students were trained to provide constructive feedback and guided on how to use a standardized rubric for reviewing reflective writings. A standardized rubric was developed after an intensive literature review. The training aimed to establish a consistent understanding of the reflective process and ensure that students were equipped with the skills necessary for meaningful peer evaluation.

Reflective writing assignment: Each student was tasked with writing a reflection on a topic covered during their clinical posting in the Community Medicine course, guided by Gibbs' Reflective Cycle. This reflective writing assignment encouraged students to systematically explore their learning experiences through the five stages (Gibbs' Reflective Cycle). The students were asked to document their recent learning in terms of description, feelings, evaluation, analysis, conclusion, and action planning.

Peer review process: Students were paired with peers for the review process, and reflective writings were exchanged for evaluation. Using a standardized peer-review rubric based on Gibbs' model, each peer provided feedback on their partner’s reflection, focusing on key aspects such as the clarity of the writing, the depth of reflection, and adherence to the stages of the reflective cycle. After receiving peer feedback, students revised their reflective writings and submitted final versions. Alongside these revisions, students included a brief reflection on how the feedback influenced their revisions, fostering deeper self-awareness about the value of constructive critique.

Data collection

Quantitative data: To assess the impact of the intervention, two types of quantitative data were collected: 1. Pre- and Post-Intervention Surveys: to measure changes in students’ perceptions regarding reflective writing and peer review. A structured questionnaire using a Likert scale (e.g., 1 = strongly disagree to 5 = strongly agree). The domains assessed were the perceived value of reflective writing, confidence in reflective writing, and understanding of peer review processes. Pre-intervention: This survey was administered before students received training on Gibbs’ Reflective Cycle and peer review. Post-intervention: This survey was administered after completing the training session on Gibb’s reflective cycle, the reflective writing and peer feedback cycle. 2. Rubric-Based Assessment of Reflective Writings: This evaluates the quality and improvement of students’ reflective writing. A standardized rubric was developed after an intensive literature review. This was introduced during the training sessions.

Key components evaluated were (a) clarity of writing (e.g., coherence and articulation of thoughts), (b) depth of reflection (e.g., critical analysis, insight), and (c) adherence to Gibbs’ Reflective Cycle (covering description, feelings, evaluation, analysis, conclusion, and action planning)

Scoring

Each domain was rated on a numerical scale (1 to 5). Two reflective samples per student were evaluated: (a) Initial submission and (b) revised version (post-peer review). Qualitative data: Focus group discussions (FGDs) were conducted to explore participants’ experiences, perceptions, and challenges related to the peer review process. Semi-structured interview guides were used to facilitate these discussions, allowing for flexibility while ensuring all relevant topics were addressed.

Data analysis

Descriptive statistics were used to summarize the survey responses. Paired t-tests were employed to compare pre- and post-intervention survey scores, while Wilcoxon signed-rank tests were applied when data distributions deviated from normality. Reflection quality scores for initial and revised submissions were also analyzed to measure improvements attributable to peer feedback. Thematic analysis was performed on the FGD transcripts to identify recurring themes and patterns in the data. This involved coding the transcripts, grouping codes into categories, and refining these into overarching themes that captured students’ experiences and perceptions of the peer review process.

## Results

Participant demographics

A total of 30 second-year medical students enrolled in the Community Medicine block posting course at Government Thoothukudi Medical College participated in the study. All 30 students completed all components of the study, including the training session, reflective writing assignments, and peer review process. The mean age of participants was 20.3 years (SD ± 1.2), with 65% identifying as female (n = 19) and 35% as male (n = 11). Participants had varying levels of prior experience with reflective writing, with 78% (n = 23) indicating they had minimal or no prior training in reflective writing (Table 1).

**Table 1:** Baseline participant demographics

Parameter	Total students enrolled n=30 (%)
Age in years (Mean ± SD)	20.3 ± 1.2
Gender distribution	
Female	19 (65%)
Male	11 (35%)
Prior experience with reflective writing	
Minimal/none	23 (78%)
Some experience	7 (22%)

Quantitative results

Pre- and Post-Intervention Surveys Results

The pre- and post-intervention surveys captured significant changes in students' perceptions and confidence regarding reflective writing. These findings highlight the impact of the peer-reviewed reflective writing intervention. Perceived Value of Reflective Writing: (n=20) 68% of students considered reflective writing a moderately valuable learning tool before the intervention. This perception shifted positively post-intervention, with the mean score for perceived value increasing from 3.2 (SD ± 0.9) to 4.5 (SD ± 0.7) on a 5-point Likert scale, demonstrating a statistically significant improvement (p < 0.001). Confidence in Reflective Writing: Prior to the intervention, only 45% of students felt confident in their ability to engage in reflective practices effectively. Post-intervention, this confidence markedly improved, with the mean score rising from 2.8 (SD ± 1.0) to 4.3 (SD ± 0.8) on a 5-point Likert scale (p < 0.001) (Table 2).

**Table 2:** Pre- and post-intervention survey results Pre-intervention mean  (SD): The average score (± standard deviation) for each domain before revision; Post-intervention mean (SD): The average score (± standard deviation) for each domain after revision; t-value: The result of a paired-sample t-test comparing initial and revised scores, indicating the magnitude and direction of change; p-value:  p-value of < 0.001 indicates a statistically significant improvement.

Measure	Pre-Intervention Mean (SD)	Post-Intervention Mean (SD)	t value	p-value
Perceived Value of Reflective Writing	3.2 ± 0.9	4.5 ± 0.7	7.67	< 0.001
Confidence in Reflective Writing	2.8 ± 1.0	4.3 ± 0.8	6.15	< 0.001

Quality of reflective writing

The quality of reflective writings was evaluated using a standardized rubric that assessed three key domains: clarity of writing, depth of reflection, and adherence to Gibbs' Reflective Cycle (Table 3). Comparisons of the initial and revised submissions demonstrated significant improvements in all assessed domains following the peer review process. The clarity of reflective writings improved significantly, with scores increasing from a mean of 3.1 (SD ± 0.8) to 4.6 (SD ± 0.5) out of 5 (p < 0.001). Students’ revisions demonstrated better organization, coherence, and articulation of their reflections. Depth of reflection, which captured students’ ability to analyze and derive insights from their experiences critically, showed a substantial improvement, with scores rising from 2.9 (SD ± 1.0) to 4.4 (SD ± 0.6) out of 5 (p < 0.001). Feedback received during the peer review process prompted more comprehensive engagement with the reflective process. Adherence to Gibbs' Reflective Cycle improved markedly, with scores increasing from a mean of 3.0 (SD ± 0.7) to 4.7 (SD ± 0.4) out of 5 (p < 0.001). Revised submissions demonstrated a more consistent and thorough application of the six stages of the reflective cycle, indicating better structural alignment with the model.

**Table 3:** Quality of reflective writing Domain: The specific aspect of reflective writing assessed, including clarity of writing, depth of reflection, and adherence to Gibbs' Reflective Cycle; Initial Mean (SD): The average score and standard deviation for each domain prior to the peer review process; Revised Mean (SD): The average score and standard deviation for each domain after the peer review process; Statistical analysis was conducted using a paired-samples t-test to compare pre- and post-intervention means. The reported t-values reflect the magnitude of change between conditions, and all differences were statistically significant with p-values less than 0.001; p-value: Statistical significance of the difference between initial and revised scores; values less than 0.001 indicate a highly significant improvement.

Domain	Initial Mean (SD)	Revised Mean (SD)	t-value	p-value
Clarity of Writing	3.1 (± 0.8)	4.6 (± 0.5)	9.00	< 0.001
Depth of Reflection	2.9 (± 1.0)	4.4 (± 0.6)	6.19	< 0.001
Adherence to Gibbs' Reflective Cycle	3.0 (± 0.7)	4.7 (± 0.4)	11.85	< 0.001

Perception of peer review

**Figure 1:**
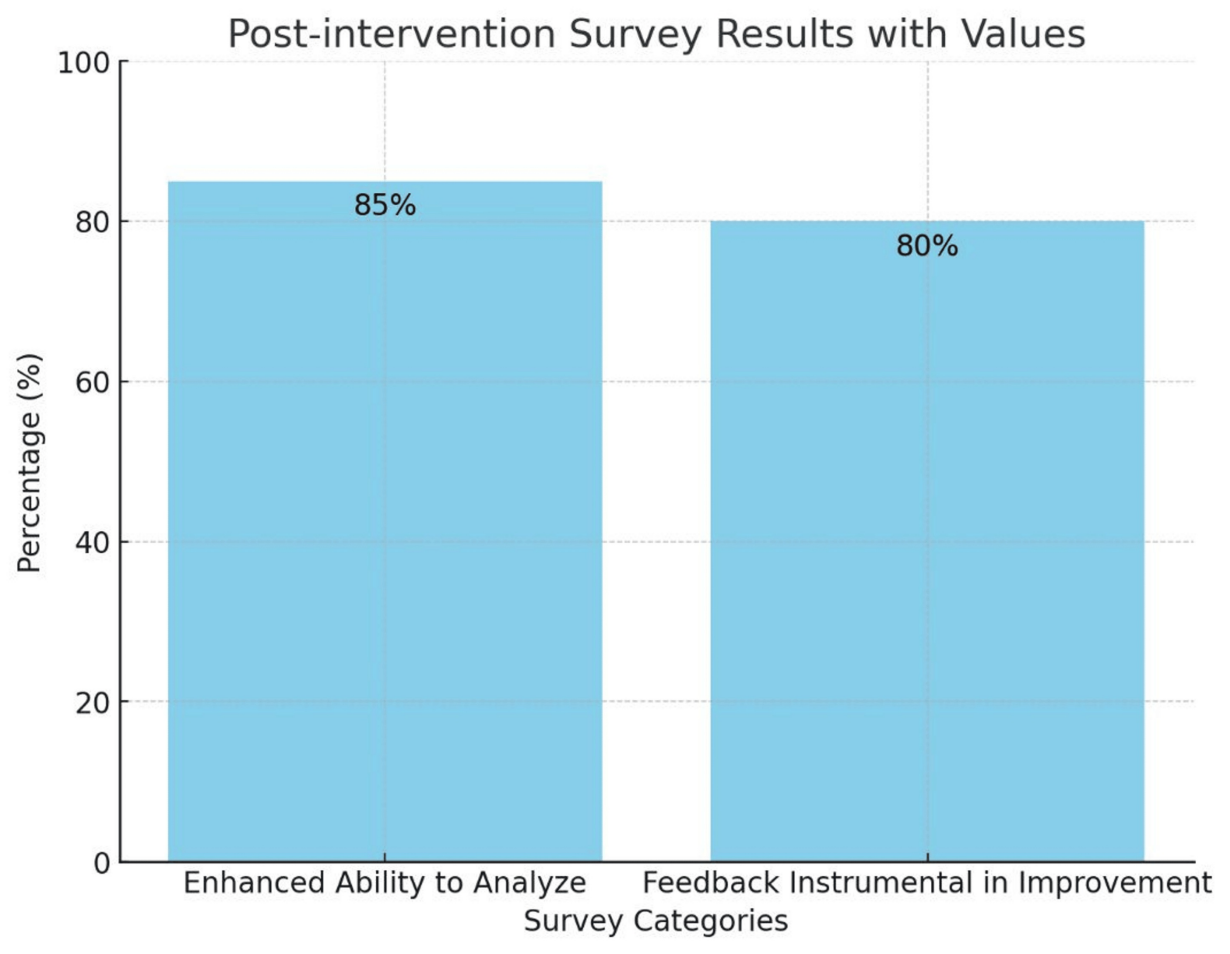
Perception of peer review Enhanced ability to analyze (85%): Percentage of students who reported an improved capacity to critically analyze and reflect on their experiences following the intervention; Feedback Instrumental in Improvement (80%): Percentage of students who perceived peer feedback as a significant contributor to the enhancement of their reflective writing skills; Y-axis (Percentage %): Represents the proportion of students endorsing each survey category; X-axis (Survey Categories): Displays the specific areas assessed in the post-intervention survey related to perceived learning outcomes.

Post-intervention surveys indicated that 85% (n=26) of students agreed that the peer review process enhanced their ability to analyze reflective writings critically, and 80% (n=24) reported that feedback received from peers was instrumental in improving their work (Figure 1).

Qualitative results

Experiences with Peer Review

**Figure 2:**
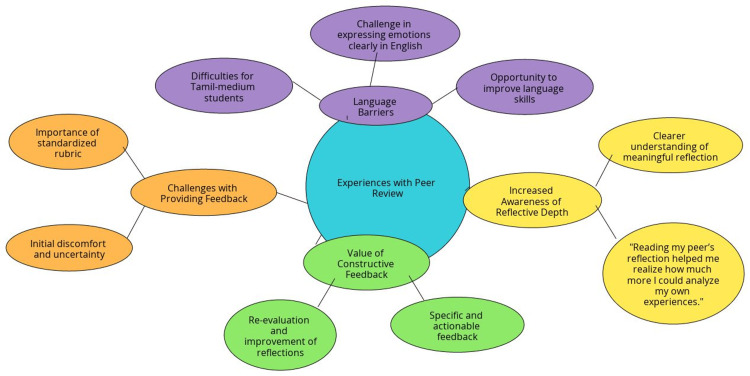
Experiences with peer review This figure was created by the authors using the collected data. Core themes and sub-themes are represented. Thematic analysis: Focal Group Discussion till the  point of saturation of data among study participants.

Thematic analysis of focus group discussions (FGDs) revealed several key themes: Increased Awareness of Reflective Depth: Students reported gaining a clearer understanding of what constitutes a deep and meaningful reflection. One participant noted, “Reading my peer’s reflection helped me realize how much more I could analyze my own experiences.” Value of Constructive Feedback: Almost all the students appreciated receiving specific and actionable feedback. Many reported that feedback prompted them to re-evaluate their initial thoughts and improve their reflections. Challenges with Providing Feedback: Some students expressed initial discomfort and uncertainty in critiquing their peers’ work. However, they acknowledged that the standardized rubric provided much-needed structure and clarity. Language Barriers: Three students from Tamil-medium educational backgrounds highlighted difficulties in reflective writing due to limited proficiency in English. They expressed that while the reflective process was intellectually stimulating, writing their reflections in English posed significant challenges. One student shared, “I found it hard to express my thoughts as deeply in English as I could in Tamil. Peer feedback helped me, but it was still tough to convey emotions clearly.” Despite these challenges, these students valued the opportunity to improve their language skills through the activity (Figure 2).

Impact on Learning and Professional Development

Students highlighted the value of peer review in fostering collaborative learning. A recurring sentiment was that the process mirrored real-world clinical practices where feedback is integral. One participant stated, “The peer review process taught me not just to reflect but also how to provide constructive feedback, which is crucial in teamwork.”

Barriers and Challenges

**Figure 3:**
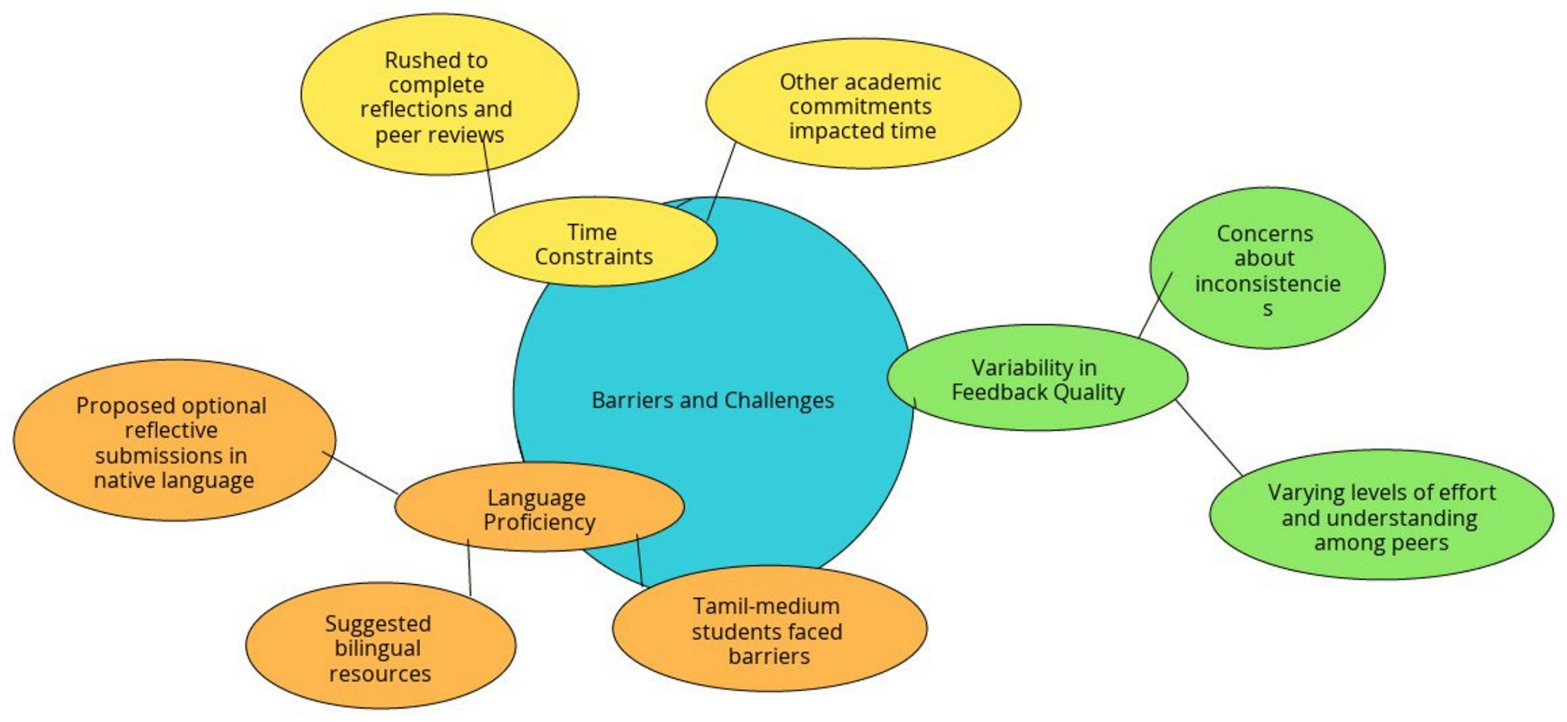
Barriers and challenges faced This figure has been created by the authors. The collected details have been compiled to enhance the visual understanding for the readers. A myriad of feedback was received from study participants during the study period.

While the majority of students found the process beneficial, a few challenges were noted: (a) Time constraints: some students reported feeling rushed to complete both their reflections and the peer reviews due to other academic commitments. (b) Variability in feedback quality: a small number of participants expressed concerns about inconsistencies in the quality of feedback they received, citing varying levels of effort and understanding among peers. (c) Language Proficiency: Students from Tamil-medium backgrounds reported additional barriers in articulating their reflections effectively. They suggested that the inclusion of bilingual resources or optional reflective submissions in their native language might enhance accessibility for students with similar challenges (Figure 3).

## Discussion

The results of this study demonstrate the effectiveness of peer-reviewed reflective writing in enhancing students’ reflective practices and fostering critical thinking, collaboration, and professional development. The findings indicate significant quantitative improvements in the quality of reflective writings, coupled with valuable qualitative insights into students' experiences with the intervention.

The pre- and post-intervention survey results highlight a substantial increase in students’ perceived value of reflective writing as a learning tool and their confidence in engaging with it. The post-intervention increase in mean scores for perceived value (from 3.2 to 4.5) and confidence (from 2.8 to 4.3) demonstrates the positive impact of structured guidance and the peer review process. These improvements align with existing literature that emphasizes the role of feedback in enhancing learners’ confidence and perceived competence in reflective practices [1,3]. The intervention significantly improved the clarity, depth, and structural rigor of reflective writings. Scores for clarity of writing increased from 3.1 to 4.6, depth of reflection from 2.9 to 4.4, and adherence to Gibbs' Reflective Cycle from 3.0 to 4.7, all statistically significant improvements (p < 0.001). These findings suggest that peer feedback played a pivotal role in helping students identify gaps in their initial reflections and refine their submissions accordingly. The structured rubric provided a consistent framework that enabled students to systematically analyze their peers’ work, which in turn reinforced their own understanding of reflective practices.

The thematic analysis revealed that students appreciated the peer review process for enhancing their ability to critically analyze reflective writings and provide constructive feedback. This aligns with the professional competencies emphasized in medical education, such as collaboration, critical thinking, and lifelong learning [4]. Students reported that engaging with their peers’ reflections broadened their perspectives and encouraged deeper engagement with their own experiences. Additionally, the process mirrored real-world clinical settings where teamwork and feedback are integral to professional growth.

Despite the overwhelmingly positive outcomes, several challenges emerged, offering insights for future interventions. Time constraints were a recurrent theme, with students indicating that balancing reflective writing and peer review with other academic responsibilities was difficult. Variability in the quality of feedback provided by peers was another challenge, highlighting the need for additional training or calibration sessions to ensure consistency in the review process. Language barriers were particularly significant for students from Tamil-medium backgrounds, who expressed difficulty in articulating their reflections in English. While these students valued the opportunity to improve their language skills, they suggested that bilingual resources or the option to write in their native language could enhance accessibility and inclusivity. Addressing these barriers is crucial for maximizing the benefits of reflective writing interventions in diverse student populations.

A 2021 qualitative meta-synthesis by Wald et al. examined health professionals' and students' experiences with reflective writing [12]. The study found that reflective writing fosters reflection and reflexivity, leading to skills development, professional growth, and increased empathy. Participants reported that reflective writing allowed them to learn from their mistakes and successes, contributing to a deeper understanding of their professional roles. A 2023 systematic scoping review by Nguyen et al. analyzed the use of reflective writing in medical education [15]. The review emphasized the importance of structured approaches, such as using standardized rubrics and guided feedback, to enhance the effectiveness of reflective writing. It also highlighted the role of peer review in providing diverse perspectives, which enriches the reflective process. A 2024 study by Kallioinen et al. investigated the reflective capacity of medical students during a general practice course [16]. The research identified that students from non-English-speaking backgrounds faced challenges in articulating their reflections due to language barriers. The study suggested that providing support, such as language resources or the option to write reflections in their native language, could enhance the reflective experience for these students. A 2020 article by Archer JC discussed the implications of grading reflective writing in medical education [17]. The authors argued that while grading can motivate students to engage seriously with reflective tasks, it may also lead to inauthentic reflections if students write what they believe assessors want to hear. The article recommended careful consideration of assessment methods to preserve the authenticity of reflective writing. A 2024 systematic scoping review by Mead et al. explored group reflection in medical education [18]. The study found that group reflective practices, including peer review, promote collaborative learning and critical thinking. Participants reported that discussing reflections with peers provided new insights and enhanced their understanding of clinical experiences.

The findings of this study have important implications for the integration of reflective writing and peer review in medical curricula. The structured approach adopted in this intervention-training students in Gibbs' Reflective Cycle, using a standardized rubric, and facilitating peer feedback-proved effective in improving students’ reflective practices. These results support the inclusion of similar frameworks in medical education to cultivate critical thinking, emotional intelligence, and collaborative learning skills. The challenges identified also highlight areas for improvement. Institutions could consider offering additional time for reflective exercises, providing resources in multiple languages, and conducting training sessions to standardize peer feedback quality. These measures could enhance the effectiveness and inclusivity of reflective writing interventions.

This study was limited to a single cohort of second-year medical students in a specific educational setting, which may limit the generalizability of the findings. Additionally, the relatively short duration of the intervention may not capture the long-term impact of peer-reviewed reflective writing on professional development. Future research could explore longitudinal effects, include diverse cohorts, and examine the use of bilingual resources to address language-related challenges.

## Conclusions

This study highlights the significant benefits of incorporating peer-reviewed reflective writing into medical education. The findings demonstrate that a structured approach, using Gibbs’ Reflective Cycle and guided peer feedback, leads to substantial improvements in the quality of students’ reflective writings, including clarity, depth, and adherence to reflective frameworks. Furthermore, the process fosters critical thinking, self-awareness, and professional development while promoting collaborative learning and the ability to give and receive constructive feedback. Despite these positive outcomes, challenges such as time constraints, variability in feedback quality, and language barriers for non-native English-speaking students were identified. Addressing these challenges through tailored interventions, such as bilingual resources, extended timelines, and enhanced peer review training, can further optimize the effectiveness of reflective writing initiatives. The study highlights the importance of reflective practice in medical education as a tool for preparing students to navigate the complexities of clinical practice. By fostering a deeper understanding of their experiences and professional roles, reflective writing equips students with essential skills for lifelong learning and compassionate patient care. Future research should explore longitudinal impacts, inclusivity measures, and scalability of peer-reviewed reflective writing programs across diverse educational settings.

## Appendices

**Table 4:** Research questionnaire administered to study group The questionnaire was developed after a rigorous literature review. A pilot study has been conducted, and the questionnaire has been standardized. The research question has been administered to the study group.

Section	Question	Response Options
1. General Understanding of Reflective Writing	How familiar are you with the concept of reflective writing?	- Very familiar, - Somewhat familiar, - Not familiar at all
1. General Understanding of Reflective Writing	What do you believe is the main purpose of reflective writing? (Select all that apply)	- Enhancing critical thinking, - Improving writing skills, - Self-awareness and personal growth, - Connecting theory to practice, - Other (specify)
1. General Understanding of Reflective Writing	How would you define reflective writing in your own words?	Open-ended
2. Experience with Reflective Writing	How often do you engage in reflective writing?	- Very often, - Occasionally, - Rarely, - Never
2. Experience with Reflective Writing	What types of reflective writing have you engaged with?	- Journals/Diaries, - Essays/Reports, - Blogs, - Creative Writing, - Other (specify)
2. Experience with Reflective Writing	What topics do you typically reflect on?	- Academic learning, - Personal experiences, - Professional development, - Social/cultural issues, - Other (specify)
2. Experience with Reflective Writing	How do you feel about reflective writing?	- Very positive, - Positive, - Neutral, - Negative, - Very negative
2. Experience with Reflective Writing	Time usually spent on reflective writing task	- < 30 minutes, - 30 mins to 1 hour, - 1–2 hours, - > 2 hours
3. Impact and Benefits	Reflective writing has improved critical thinking	- Very much, - Somewhat, - Not much, - Not at all
3. Impact and Benefits	Impact on academic performance	- Significant improvement, - Moderate, - No impact, - Negative
3. Impact and Benefits	Helped self-awareness and personal growth	Likert scale: Strongly agree → Strongly disagree
3. Impact and Benefits	Beneficial for future career	- Very beneficial, - Somewhat, - Not beneficial, - Not sure
3. Impact and Benefits	Helped connect academic learning to real-world	Likert scale: Strongly agree → Strongly disagree
4. Challenges and Obstacles	Challenges faced in reflective writing	- Lack of time, - Difficulty organizing thoughts, - Uncertainty on how to write reflectively, - Lack of motivation, - Other (specify)
4. Challenges and Obstacles	Are task expectations clear?	- Very clear, - Clear, - Unclear, - Very unclear
4. Challenges and Obstacles	Usual approach to reflective writing	- Freely and spontaneously, - Structured outline, - Plan and organize first, - Other (specify)
4. Challenges and Obstacles	Is it more time-consuming than other academic tasks?	- Yes, significantly, - Yes, somewhat, - No, about the same, - No, less time
5. Perceptions and Recommendations	How could reflective writing be improved?	Open-ended
5. Perceptions and Recommendations	Would you recommend reflective writing?	- Yes, definitely, - Yes, with reservations, - No
5. Perceptions and Recommendations	What support would help improve reflective writing?	- Guides/templates, - Workshops, - Instructor feedback, - Peer review, - Other (specify)
Closing	Anything else you'd like to share?	Open-ended
